# A quantitative 14-3-3 interaction screen connects the nuclear exosome targeting complex to the DNA damage response

**DOI:** 10.1101/gad.246272.114

**Published:** 2014-09-15

**Authors:** Melanie Blasius, Sebastian A. Wagner, Chunaram Choudhary, Jiri Bartek, Stephen P. Jackson

**Affiliations:** 1 The Gurdon Institute,; 2 Department of Biochemistry, University of Cambridge, Cambridge CB2 1QN, United Kingdom;; 3 Genome Integrity Unit, Danish Cancer Society Research Centre, 2100 Copenhagen, Denmark;; 4 The Novo Nordisk Foundation Center for Protein Research, Faculty of Health and Medical Sciences, University of Copenhagen, 2200 Copenhagen, Denmark;; 5 Institute of Molecular and Translational Medicine, Palacky University, 77900 Olomouc, Czech Republic;; 6 The Wellcome Trust Sanger Institute, Hinxton, Cambridge CB10 1SA, United Kingdom

**Keywords:** DNA damage response, 14-3-3, nuclear exosome, MAPKAPK2, UV

## Abstract

RNA metabolism is altered following DNA damage, but the underlying mechanisms are not well understood. Through a 14-3-3 interaction screen for DNA damage-induced protein interactions in human cells, we identified protein complexes connected to RNA biology. These include the nuclear exosome targeting (NEXT) complex that regulates turnover of noncoding RNAs termed promoter upstream transcripts (PROMPTs). We show that the NEXT subunit RBM7 is phosphorylated upon DNA damage by the MAPKAPK2 kinase and establish that this mediates 14-3-3 binding and decreases PROMPT binding. These findings and our observation that cells lacking RBM7 display DNA damage hypersensitivity link PROMPT turnover to the DNA damage response.

## Abstract (precis)

Through a 14-3-3-interaction screen for DNA damage-induced protein interactions in human cells, Blasius et al. identified protein complexes connected to RNA biology. These include the nuclear exosome targeting (NEXT) complex that regulates turnover of noncoding RNAs termed promoter upstream transcripts (PROMPTs). The NEXT subunit RBM7 is phosphorylated upon DNA damage by the MAPKAPK2 kinase, and this mediates 14-3-3 binding and decreases PROMPT binding.

The cellular DNA damage response (DDR) is controlled by protein post-translational modifications, particularly phosphorylation (Polo and Jackson 2011). Key DDR protein kinases are ataxia telangiectasia-mutated (ATM) and ataxia- and Rad3-related (ATR) that phosphorylate and activate the DDR kinases CHK2 and CHK1, respectively (Jackson and Bartek 2009; Smith et al. 2010). Another aspect of the DDR is activation of p38^MAPK^ and MAPKAP kinase-2 (MK2)-dependent signaling (Bulavin et al. 2001; Reinhardt and Yaffe 2009), which includes MK2-mediated phosphorylation of RNA-binding proteins (Reinhardt et al. 2010). Notably, the consensus target motifs for CHK1, CHK2, and MK2 (Yaffe et al. 1997; Blasius et al. 2011) resemble phospho-dependent binding sites for 14-3-3 proteins (Johnson et al. 2011)—highly conserved phospho-binding factors that are encoded by seven human genes, form homodimers and heterodimers, and regulate protein interactions, conformations, and localizations (Mohammad and Yaffe 2009). Highlighting DDR roles for 14-3-3 proteins, DNA damage-induced CHK1 phosphorylation triggers 14-3-3 binding and CHK1 nuclear accumulation (Chen et al. 1999; Jiang et al. 2003).

Transcription occurs ∼0.5–2.5 kb upstream of mammalian RNA polymerase I, II, and III promoters, producing promoter upstream transcripts (PROMPTs) (Preker et al. 2008, 2011). PROMPTs are degraded by the RNA exosome (Chlebowski et al. 2013), and because exosome depletion caused changes in promoter DNA methylation, it is proposed that PROMPTs influence this methylation to alter gene expression (Preker et al. 2008). Recently, the human nuclear exosome targeting (NEXT) complex was shown to mediate PROMPT degradation by delivering them to the RNA exosome (Lubas et al. 2011). NEXT comprises RBM7, which contains an RNA-binding motif (Guo et al. 2003); ZCCHC8 (Gustafson et al. 2005); and the putative RNA helicase MTR4/SKIV2L2. The precise functions for PROMPTs and the NEXT complex and whether they are regulated remain unknown. Here, we show that ultraviolet light (UV) exposure leads to MK2-mediated RBM7 phosphorylation, triggering 14-3-3 binding and controlling PROMPT turnover, thus highlighting PROMPT regulation as a new facet of the DDR.

## Results and Discussion

### Identifying novel UV-induced 14-3-3-interacting proteins

**Figure 1.**
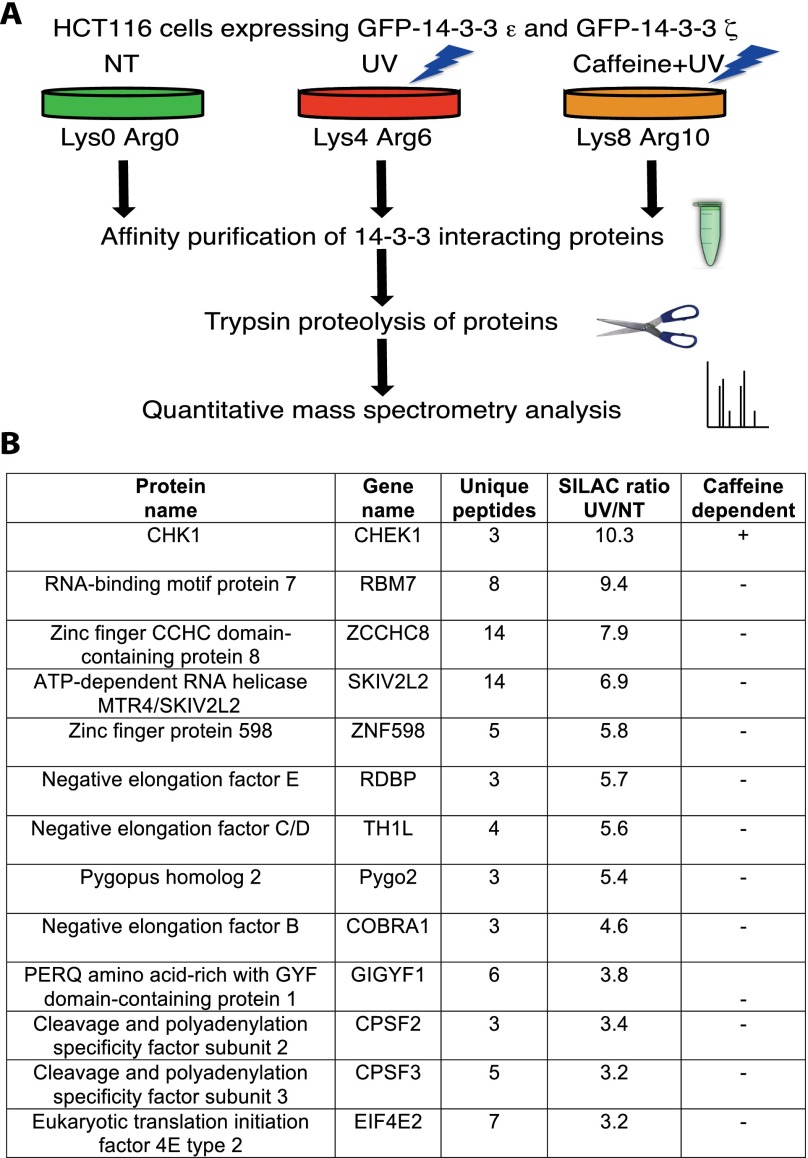
Identifying UV-induced 14-3-3-interacting proteins. (*A*) Strategy. (NT) Cells grown in “light” SILAC medium and left nontreated; (UV) cells grown in “medium” SILAC medium and treated with 40 J/m^2^ UV-C 2 h prior to harvesting; (Caffeine+UV) cells grown in “heavy” SILAC medium, pretreated for 1 h with 4 mM caffeine, and then treated with 40 J/m^2^ of UV-C 2 h prior to harvesting. (*B*) High-confidence, UV-induced 14-3-3 interactors. Selected proteins with three or more unique peptides and a more than threefold increased binding are shown. (SILAC ratio UV/NT) Fold increased 14-3-3 binding upon UV compared with nontreated cells; (caffeine dependence) UV-induced binding was reduced at more than threefold by caffeine (+) or less than twofold (−).

We performed a quantitative proteomic screen to identify UV-induced 14-3-3 interactors (Fig. 1A) by cotransfecting human HCT116 cells with plasmids expressing green fluorescent protein (GFP)-tagged 14-3-3ɛ and 14-3-3ζ, two of the seven human 14-3-3 isoforms with links to cell cycle control via binding CHK1 and CDC25 (Jiang et al. 2003; Dalal et al. 2004). GFP-14-3-3-expressing cells were grown in SILAC (stable isotope labeling by amino acids in cell culture) medium (Ong et al. 2002) and then treated or not with UV in the presence or absence of caffeine, an ATM/ATR inhibitor. Ensuing GFP-14-3-3 isolation and mass spectrometry (MS) identified 21 proteins whose binding was increased more than threefold by UV (Fig. 1B; Supplemental Table 1).

The protein in our list with the highest UV-induced 14-3-3 binding was CHK1, a well-characterized UV-induced 14-3-3 interactor (Chen et al. 1999; Jiang et al. 2003), and this was the only strong hit whose UV-induced binding was more than threefold reduced by caffeine. We also identified proteins with reduced 14-3-3 binding upon UV (Supplemental Table 1), including BAD, a proapoptotic protein dephosphorylated and released from 14-3-3 proteins after UV (Sunayama 2005), and SRp38, a splicing regulator dephosphorylated upon stress, leading to decreased 14-3-3 binding and splicing inhibition (Shi and Manley 2007). Strikingly, our list of UV-induced 14-3-3 interactors was dominated by proteins connected with RNA (Fig. 1B). These included the NEXT complex subunits RBM7, ZCCHC8, and MTR4 (Lubas et al. 2011); the CPSF2 and CPSF3 components of the mRNA cleavage and polyadenylation complex (CPSF) (Darnell 2013); the EIF4E and ZNF598 subunits of the 4EHP–GIGYF complex involved in translational repression (Morita et al. 2012); and the NELF-E, NELF-C/D, and NELF-B subunits of the NELF complex that controls transcription elongation (Nechaev and Adelman 2011).

### Validation of RBM7 as a UV-induced 14-3-3 interactor

**Figure 2.**
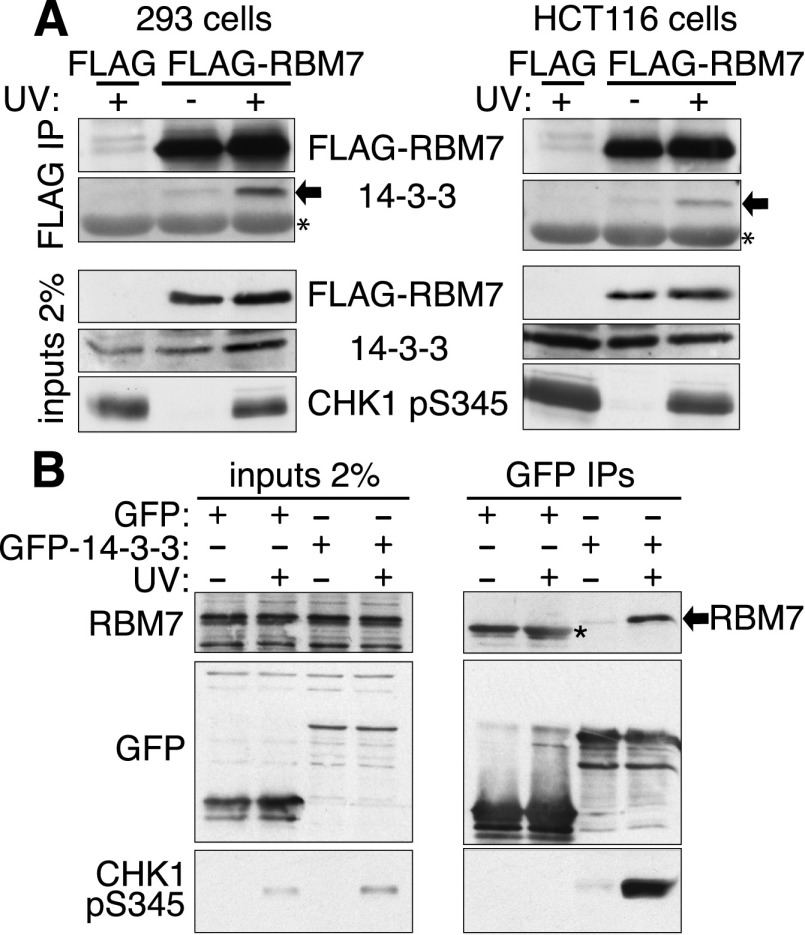
Interaction between 14-3-3 and RBM7. (*A*) Increased interaction (arrows) after UV in 293 and HCT116 cells, shown by Flag immunoprecipitation. (NT) Nontreated; (UV) cells harvested 2 h post UV-C. The asterisk indicates a cross-reacting band, and the arrow indicates 14-3-3. (*B*) GFP-14-3-3 proteins were expressed in HCT116 cells and immunoprecipitated from nontreated or UV-treated cells. The asterisk indicates unspecific bands resulting from GFP protein.

The protein with the strongest UV-induced 14-3-3-binding ratio after CHK1 was RBM7. Because RBM7 is phosphorylated on multiple sites (Hornbeck et al. 2004) and since 14-3-3 proteins function as dimers that can bind paired target protein phosphorylations (Johnson et al. 2010), we focused on RBM7 as a potential direct 14-3-3 interactor. In accord with our MS data, Flag-tagged RBM7 interacted with endogenous 14-3-3 proteins in a UV-dependent manner in human 293 and HCT116 cells (Fig. 2A). Furthermore, reciprocal binding studies with bacterially expressed GST-14-3-3 (Supplemental Fig. S1A) revealed increased interaction between 14-3-3 and Flag-RBM7 upon UV exposure in 293 and HCT116 cells (Supplemental Fig. S1B). By raising an antiserum against RBM7 (Supplemental Fig. S1C), we established that endogenous RBM7 coimmunoprecipitated with 14-3-3 proteins in a UV-dependent manner (Fig. 2B). Also, through expressing GFP or GFP-RBM7 in cells, immunoprecipitation, and identifying interactors by MS, we established that all 14-3-3 isoforms specifically interacted with RBM7, with 14-3-3ζ and 14-3-3ɛ showing the most unique peptides and the highest GFP-RBM7:GFP SILAC ratios (Supplemental Tables 2, 3). In parallel immunoprecipitation studies with GFP-RBM7, we established that NEXT components ZCCHC8 and MTR4 were RBM7-associated irrespective of UV treatment (Supplemental Fig. S1D). Also, no changes in GFP-RBM7 subcellular localization were seen after UV-C irradiation (Supplemental Fig. S2D,E, note that RBM7 did not appear to accumulate in UV-induced mRNA granules that may arise via RNA damage; Gaillard and Aguilera 2008). Furthermore, our data suggested that 14-3-3 binding to other NEXT subunits was via RBM7, as RBM7 depletion prevented 14-3-3 binding by ZCCHC8 (Supplemental Fig. S1E).

### DNA damage-induced RBM7 phosphorylation mediates 14-3-3 binding

**Figure 3.**
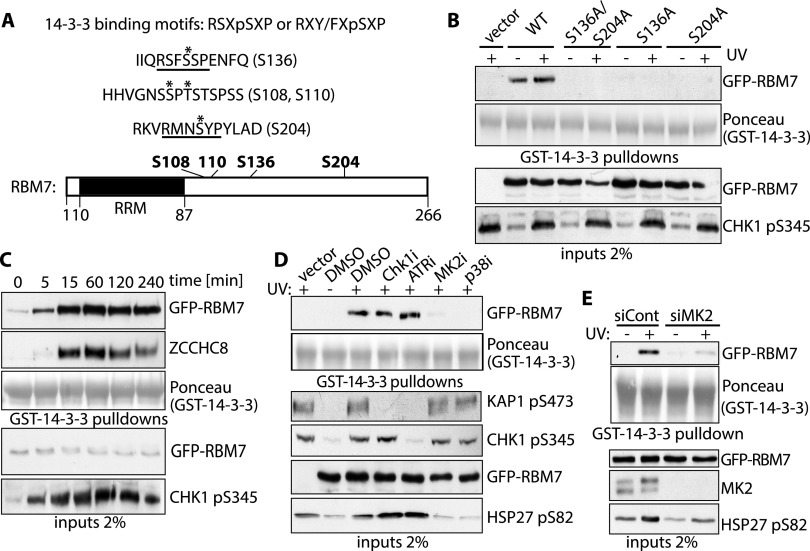
RBM7 is phosphorylated by MK2 in response to UV. (*A*, *top*) Peptides identified by phosphopeptide mapping. UV-induced phosphorylated residues are marked by an asterisk, and phosphorylated residue positions are in brackets. 14-3-3-binding motifs are underlined, and consensus 14-3-3-binding motifs are shown *above*. (*Bottom*) RBM7 domain structure. RNA recognition motif (RRM; black) and residues phosphorylated upon UV are in bold. (*B*) GST-14-3-3 pull-downs for the indicated GFP-RBM7 derivatives. (*C*) Time course of interaction between 14-3-3 and RBM7. U2OS cells stably expressing GFP-RBM7 were UV-C-treated and harvested at the indicated times, GST-14-3-3 pull-downs were done, and interactions with GFP-RBM7 and ZCCHC8 were monitored. (*D*) U2OS cells stably expressing GFP-RBM7 (lanes *2*–*7*) or empty vector (lane *1*) were UV-C-treated in the presence or absence of the indicated inhibitors, and extracts were used for GST-14-3-3 pull-down assays. (*E*) U2OS cells stably expressing GFP-RBM7 were transfected with control siRNA (siCont) or siRNA against MK2 (siMK2) and irradiated with 40 J/m^2^ UV-C 2 h prior to harvesting and GST-14-3-3 pull-downs.

RBM7 and 14-3-3 interacted even after RNase A treatment, suggesting that their interaction was direct and not mediated by RNA (Supplemental Fig. S1F). Quantitative analysis of RBM7 phosphorylation revealed that four phosphorylations increased upon cellular UV irradiation (Fig. 3A), two of which (Ser136 and Ser204) matched the 14-3-3 binding consensus (Yaffe et al. 1997). To determine whether these mediated 14-3-3 binding, we generated stable HCT116 cells expressing GFP, GFP-tagged wild-type RBM7 (RBM7-WT), GFP-RBM7-S136A, GFP-RBM7-S204A, or GFP-RBM7 with both serines mutated to alanine (S136A/S204A). We mock- or UV-treated cells, lysed them, added and retrieved GST-14-3-3, and then assessed samples for RBM7 binding. Strikingly, while RBM7-WT displayed 14-3-3 binding, none was observed for RBM7-S136A, RBM7-S204A, or RBM7-S136A/S204A (Fig. 3B), suggesting that effective 14-3-3 binding requires RBM7 phosphorylation on both Ser136 and Ser204. We observed that RBM7 and ZCCHC8 rapidly associated with 14-3-3 upon UV irradiation and that this persisted for >4 h (Fig. 3C). Because the UV-induced DDR also persists for similar time frames, as indicated by CHK1 Ser345 phosphorylation (Fig. 3C), the interaction between RBM7 and 14-3-3 likely reflects DDR-induced RBM7 phosphorylation. In parallel studies, we found that other DNA-damaging agents also induced 14-3-3 binding (Supplemental Fig. S2A).

### RBM7 interaction with 14-3-3 is mediated by the p38–MK2 pathway

Because the UV-induced RBM7 phospho sites that we identified matched CHK1 and MK2 target motifs (Yaffe et al. 1997; Blasius et al. 2011), we tested the effects of ATR, CHK1**/**2, p38, and MK2 inhibitors on 14-3-3 binding by RBM7. While p38 or MK2 inhibition essentially abrogated the UV-induced interaction between RBM7 and 14-3-3, CHK1 or ATR inhibition had no effect (Fig. 3D, phosphorylated KAP1 Ser473, CHK1 Ser345, and HSP27 Ser82 demonstrate effective CHK1 or ATR inhibition, ATR inhibition, or p38/MK2 inhibition, respectively). Because p38 is the upstream activating kinase for MK2 and since inhibitors against both abolished UV-induced interaction between RBM7 and 14-3-3, we speculated that MK2 directly targeted RBM7. Indeed, transfecting cells with an MK2 siRNA strongly reduced UV-induced interaction between RBM7 and 14-3-3 (Fig. 3E). Furthermore, in vitro phosphorylation of GFP-RBM7 by MK2 increased 14-3-3 binding (Supplemental Fig. S2C). In line with the p38–MK2 pathway also being stimulated by other stresses (Dorion and Landry 2002), interaction between RBM7 and 14-3-3 was also increased upon heat shock (Supplemental Fig. S2B).

### UV induces PROMPTs and decreases their binding to RBM7

**Figure 4.**
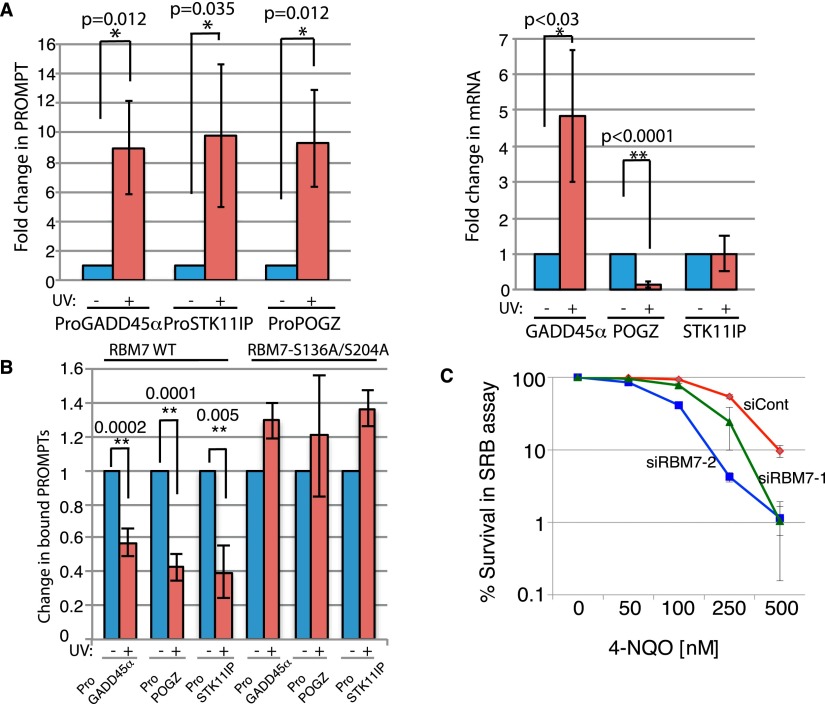
PROMPT levels are increased and RBM7–PROMPT interaction was decreased upon UV. (*A*) Levels of three representative PROMPTs (*left* panel) and their corresponding mRNAs (*right* panel), measured by quantitative PCR (qPCR) from HeLa cells that were treated or not with 40 J/m^2^ UV-C and harvested 4 h later. GADD45 mRNA induction was a readout for UV signaling. Blue bars indicate nontreated samples, and red bars indicate UV-treated samples. (*B*) HCT116 cells were transfected with Flag-RBM7 and, after 48 h, treated or not with 40 J/m^2^ UV-C 4 h prior to harvesting. RBM7-associated PROMPTs were quantified by qPCR, and PROMPT levels from nontreated (NT) cells were set as 1. (*C*) Cytotoxicity of HCT116 cells in response to chronic 4-nitroquinoline 1-oxide (4-NQO) treatment measured by SRB assay. Error bars show standard deviation from three independent experiments. (*) *P*-value < 0.1; (**) *P*-value < 0.01.

RBM7 depletion causes PROMPT accumulation (Lubas et al. 2011), suggesting that without RBM7, the NEXT complex cannot deliver PROMPTs to the RNA exosome for degradation. We reasoned that if UV-dependent RBM7 phosphorylation and 14-3-3 binding affected PROMPT targeting, PROMPT levels would change upon UV. Indeed, UV irradiation of HeLa cells markedly increased three representative PROMPTs: ProGADD45α (based on ENCODE/CSHL contig_124261), ProPOGZ, and ProSTK11IP (Fig. 4A, left; Lubas et al. 2011). Furthermore, these inductions occurred irrespective of whether corresponding mRNA levels were elevated, reduced, or unchanged (Fig. 4A, right, GADD45α, POGZ, and STK11IP, respectively). PROMPT levels also increased in U2OS cells upon RBM7 depletion or UV treatment (Supplemental Fig. S3A,B), while RBM7 depletion did not significantly change corresponding mRNAs levels (Supplemental Fig. S3A).

The above data suggested that UV-induced RBM7 phosphorylation decreases RBM7 binding to PROMPTs and targeting them to the exosome. Indeed, immunoprecipitation studies revealed that UV treatment decreased PROMPT binding to Flag-RBM7 but not Flag-RBM7-S136A/S204A (binding to FLAG-RBM7-S136A/S204A actually increased, likely reflecting PROMPT induction after UV) (Fig. 4B). Similar effects were observed when we used GFP-tagged RBM7 constructs (Supplemental Fig. S3C). In line with these findings, overexpression of RBM7-S136A/S204A but not RBM7-WT prevented UV-induced PROMPT accumulation (Supplemental Fig. S3E). Furthermore, consistent with these RBM7-mediated responses playing a functional role in response to DNA damage, RBM7 depletion by two different siRNAs caused hypersensitivity to the UV-mimicking drug 4-nitroquinoline 1-oxide (4-NQO) (Fig. 4C). As shown in Supplemental Figure S3D, 4-NQO hypersensitivity was also seen upon RBM7 depletion in U2OS cells and was partially rescued by expression of GFP-RBM7-WT. In contrast, expression of RBM7-S136A/S204A, which is not controlled by MK2, increased cell survival, suggesting that lower PROMPT levels promote survival of damaged cells.

**Figure 5.**
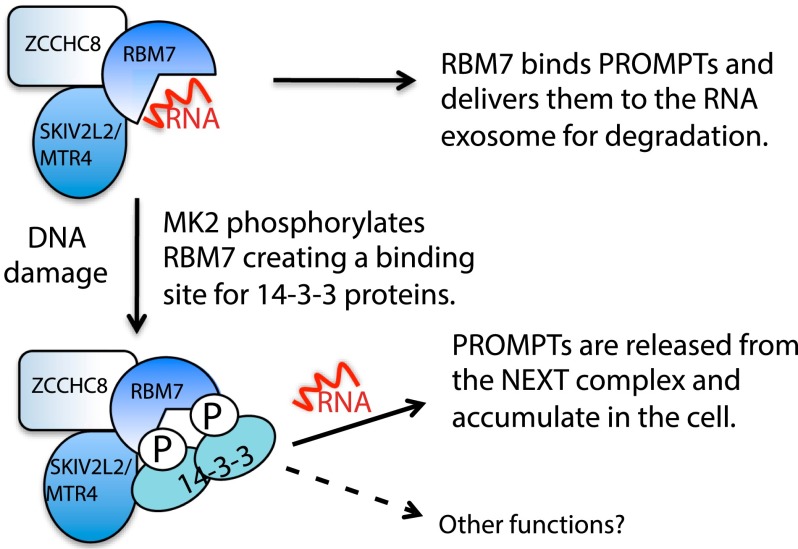
Model for NEXT regulation in response to UV.

In summary, through proteomic screening, we identified various factors whose interactions with 14-3-3 proteins are altered by UV exposure. Although we focused follow-up work on pathways connected to the DDR, we note that RNA damage could also contribute to the responses that we observed. It is striking that the majority of proteins identified displaying the most pronounced UV-induced changes in 14-3-3 binding have intimate connections to RNA. Our data are thus in line with other work (Sette 2010; Reinhardt et al. 2011; Beli et al. 2012; Bhatia et al. 2014; Britton et al. 2014) identifying RNA-associated proteins as impacting on the DDR, highlighting how controlling RNA metabolism and functions is likely to represent important but as-yet relatively unexplored aspects of the DDR. Our findings also suggest new linkages between responses mediated by p38/MK2 and events such as RNA polyadenylation, transcriptional elongation, and translational control that could now be explored. Exemplifying the potential for such work, our studies on the NEXT component RBM7 has led to a model in which RBM7 binds PROMPTs in unchallenged cells, targeting them for degradation by the nuclear RNA exosome. Upon DNA (and potentially RNA) damage created by UV or other stresses, MK2 phosphorylates RBM7 on Ser136 and Ser204, creating a binding site for 14-3-3 proteins that impairs RBM7 RNA binding, preventing the NEXT complex from delivering PROMPTs and possibly other RNAs for degradation. Consequently, PROMPT levels increase upon UV, potentially enhancing cell survival via changes in gene expression (Fig. 5). It will be interesting to see whether RBM7 is involved in other aspects of RNA metabolism. Indeed, as RBM7 also interacts with splicing factors and the nuclear proteasome (Supplemental Tables 2, 3; Lubas et al. 2011), it will be worthwhile assessing whether these interactions are affected by MK2-mediated RBM7 phosphorylation and 14-3-3 binding. Additionally, it will be interesting to establish how RBM7 phosphorylation and 14-3-3 interactions affect PROMPT binding and how UV-induced changes in PROMPT levels or other readouts of NEXT complex activity affect cell physiology.

## Materials and methods

Additional methods are described in the Supplemental Material.

### Protein–RNA coimmunoprecipitations

Pellets of cells expressing Flag- or GFP-tagged RBM7 were lysed in buffer A (50 mM Tris-HCl at pH 7.5, 100 mM NaCl, 1% NP-40, 0.1% SDS, 0.5% deoxycholate, anti-RNase [Ambion], protease inhibitor tablets, PhosSTOP [Roche]) and sonicated for 5 sec at 30% amplitude, and extracts were centrifuged for 20 min at maximum speed. Protein extract was used for GFP immunoprecipitation (GFP-Trap agarose beads [ChromoTek]) or Flag immunoprecipitation (anti-Flag M2 affinity gel [Sigma-Aldrich]) using 20 µL of beads. After 1 h at 4°C, beads were washed three times with buffer A. Retained RNA was purified by an RNeasy Mini Kit (Qiagen), reverse-transcribed with high-capacity cDNA reverse transcription kit (Applied Biosystems), and quantified by quantitative PCR. Results were quantified by ΔΔCt method, normalizing first to GAPDH and then to corresponding nontreated control.
